# Biodegradation of di-(2-ethylhexyl) phthalate by a halotolerant consortium LF

**DOI:** 10.1371/journal.pone.0204324

**Published:** 2018-10-15

**Authors:** Fangfang Li, Yidan Liu, Diwei Wang, Chaosheng Zhang, Zhihui Yang, Siqi Lu, Yangyang Wang

**Affiliations:** 1 Institute of Sustainable Development in Agriculture and Rural Area, Henan University, Kaifeng, Henan, China; 2 International Network for Environment and Health, School of Geography and Archaeology, National University of Ireland, Galway, Ireland; 3 Department of Environmental Engineering, School of Metallurgy and Environment, Central South University, Changsha, China; 4 Chinese National Engineering Research Centre for Control and Treatment of Heavy Metal Pollution, Changsha, China; 5 College of Plant Science, Jilin University, Changchun, China; 6 Key Research Institute of Yellow River Civilization and Sustainable Development & Collaborative Innovation Center on Yellow River Civilization of Henan Province, Henan University, Kaifeng, China; Babasaheb Bhimrao Ambedkar University, INDIA

## Abstract

A halotolerant bacterial consortium capable of degrading di-(2-ethylhexyl) phthalate (DEHP) was enriched from activated sludge. Community analysis revealed that LF contained seven families and seven genera of bacteria. The predominant species was *Gordonia* sp. (54.93%), *Rhodococcus*. sp. (9.92%) and *Achromobacter* sp. (8.47%). The consortium could degrade 93.84% of 1000 mg/l DEHP after 48 h incubation. The optimal temperature and pH for LF to degrade DEHP were 30 °C and 6.0, respectively. LF degraded more than 91% of DEHP with salt concentrations ranging from 0–3%. The inoculum size had great effects on DEHP degradation (incubation time < 24h). LF could degrade high concentrations of DEHP (from 100 to 2000 mg/l) with the degradation ratio above 92% after 72 h incubation. Kinetics analysis revealed that the degradation of DEHP by LF was best fitted by the first-order kinetics when the initial concentration ranged from 100 to 2000 mg/l. The main intermediates (2-ethylhexyl pentyl phthalate, butyl (2-ethylhexyl) phthalate (BEHP), mono-ethylhexyl phthalate (MEHP), mono-hexyl phthalate (MHP), mono-butyl phthalate (MBP)) in DEHP degradation process were identified using gas chromatography–mass spectrometry (GC-MS), and a new complex biochemical pathway was proposed. Furthermore, LF could also degrade dimethyl phthalate (DMP), diethyl phthalate (DEP), di-*n*-butyl phthalate (DBP), di-*n*-octyl phthalate (DOP) and phthalic acid (PA).

## Introduction

Phthalic acid esters (PAEs) is a group of teratogenicity, carcinogenicity, mutagenicity and endocrine disruption compounds, which are widely used in PVC plasticizer to enhance their plasticity and flexibility [1–3]. As they are not chemically bound to the products, PAEs can be easily released to the environment during manufacturing, usage and disposal, and consequently they are widely distributed in soils, wastewater, air, seawater, sewage sludge and sediments [4–9].

Among the PAEs, di-(2-ethylhexyl) phthalate (DEHP) is one of the most predominant used plasticizers, which has been detected in various products, such as cosmetics, toys, packaging materials, medical devices and even in human tissues [10–12]. In an investigation of 85 samples of infant umbilical cord blood, a total of 65 samples were found to contain DEHP [13]. Liu et al. [14] analyzed the contamination in two waterworks of Harbin city in Northeast China, and found that the mean concentrations of DEHP were 3473.7 ng/l and 4059.2 ng/l, respectively, which poses a great threat to human health. Therefore, it is necessary to remove DEHP from the environment economically and effectively.

Available methods to remove DEHP from the environment include hydrolysis, photolysis, adsorption and biodegradation [15–18]. Numerous researches have indicated that the degradation of DEHP by microbiology is the major route in the environment, because the hydrolysis and photolysis ratio of DEHP is very low. Recently, a variety of bacterial strains capable of degrading DEHP have been isolated from the environment, such as *Acinetobacter* sp. [19], *Microbacterium* sp. [20], *Bacillus* sp. [21], *Gordonia* sp. [22], *Pseudoxanthomonas* sp. [23] etc. However, most of these researches focused on degradation of DEHP by pure bacterial strains, and little attention has been paid to DEHP degradation by bacteria consortium or bacteria combination. Previous reports indicated that bacteria consortium or bacteria combination has a strong adaption to the adverse environment with a more efficient metabolism [24–26]. Therefore, it is necessary to investigate the biodegradation of DEHP by bacteria consortia.

Meanwhile, a large amount of salinity wastewater containing DEHP can be produced during quite a few industrial processes, such as chemicals, paint, natural gas collection and marine oil exploitation [27, 28]. Salt content is considered to be a significant factor in the biodegradation process of organic pollutants, and the growth and metabolic activity of bacteria can be strongly influenced by salinity [29, 30]. Therefore, it is important to screen a halophilic bacteria or bacteria consortium to remediate DEHP under high salinity condition. Previous studies have reported the biodegradation of DMP [31] and DBP [32] under high salinity condition, but to our knowledge, no report about DEHP has been published.

In the present study, a salt-tolerant DEHP degrading bacterial consortium was enriched from sewage treatment activated sludge. Environmental factors affecting DEHP degradation and the kinetics of DEHP removal under optimal conditions were investigated. The degradation intermediates of DEHP by bacterial consortium were also detected using gas chromatography-mass spectrometer (GC-MS).

## Materials and methods

### Chemical reagents

Di-(2-ethylhexyl) phthalate (DEHP), dimethyl phthalate (DMP), diethyl phthalate (DEP), di-*n*-butyl phthalate (DBP), and di-*n*-octyl phthalate (DOP) were purchased from Aladdin-reagent Co., Shanghai, China at 99% purity. Methanol (HPLC grade), ethyl acetate (analytical grade), and other chemical reagents (analytical grade) were purchased from the Chinese Medicine Group (Shanghai, China).

### Media and enrichment of DEHP-degrading consortium

The consortium was enriched from the sewage treatment activated sludge collected from Xinxiang, Henan Province, China. The medium used in this experiment was the minimum salt medium (MSM) with an addition of 3% extra NaCl adopted by Wu et al. [33] and named as MSMs. The enrichment procedure for the DEHP-degrading bacteria consortium was the same as our previous report [34]. The final enrichment culture was named LF which was used for further degradation experiments. The community analysis was conducted according our previous report [34].

### DEHP degradation experiments using the consortium LF

The LF was inoculated in 1000 mg/l DEHP in sterilized MSMs at 30 °C on a rotary shaker at 175 rpm. The cells were harvested after 48h and washed three times with 0.05 mol/l potassium phosphate buffer (pH 7.5), then re-suspended in the same phosphate buffer to an OD600 of 1.0. To determine the effect of environmental factors on DEHP degradation, single factor optimizations were performed including pH value (4.0, 5.0, 6.0, 7.0, 8.0, 9.0 and 10.0), temperature (20, 25, 30, 35, 40 and 45°C) and salt concentration (W/V) of NaCl (1, 2, 3, 4, 5, 6, 7, 8, 9 and 10%). The consortium at a dose of 5% was added to 50 ml Erlenmeyer flasks containing 19 ml sterilized MSMs. All the experiments were conducted with the concentration of DEHP at 1000 mg/l, on a rotary shaker of 175 rpm in the dark for 48h and performed in triplicate.

### The influence of inoculum size on DEHP degradation by LF

The effects of different inoculum size (1, 2, 3, 4, 5, 6, 7, 8, 9 and 10%, v/v) on 1000 mg/l DEHP degradation were evaluated under the optimum cultivation conditions. The experimental samples were collected at 24h and 48h and performed in triplicate.

### Kinetics of DEHP degradation by LF

Degradation kinetic experiments with different initial concentrations of DEHP (50, 100, 200, 300, 400, 500, 1000 and 2000 mg/l) by consortium LF were performed under the optimum cultivation conditions with the consortium dose of 5%. The experimental samples were collected every 12 h within 3 days and were stored at 4°C for further UPLC analysis.

### Analysis of DBP degradation intermediates

The degradation intermediates of DEHP were detected using GC–MS (Agilent, USA) and the samples were concentrated approximately 10-fold prior to GC-MS analysis. The detection procedure conditions were set according to He [35] et al.

### Substrate utilization experiments

The LF was inoculated in MSMs medium supplemented with the following substrate (400 mg/l): DMP, DEP, DBP, DOP, DEHP, and phthalic acid (PA), respectively. The potential ability of substrate utilization was assayed using microbial growth by measuring turbidity at 600 nm after 48 h incubation. All experiments and controls were performed in triplicate.

### Analytical methods

Extraction of residual DEHP and degradation intermediates from the liquid was performed following the procedure described in Wu [36] et al. The chromatographic conditions for detecting DEHP by UPLC (Waters, US) were as follows: column ACQUITY UPLC BEH C18 (2.1 mm×50 mm, 1.7 μm), mobile phase, methanol: water (90:10 [v/v]); the flow rate (0.4 ml/min); and UV wave-length (254 nm).

## Results and discussion

### Community analysis of LF

**Fig 1:**
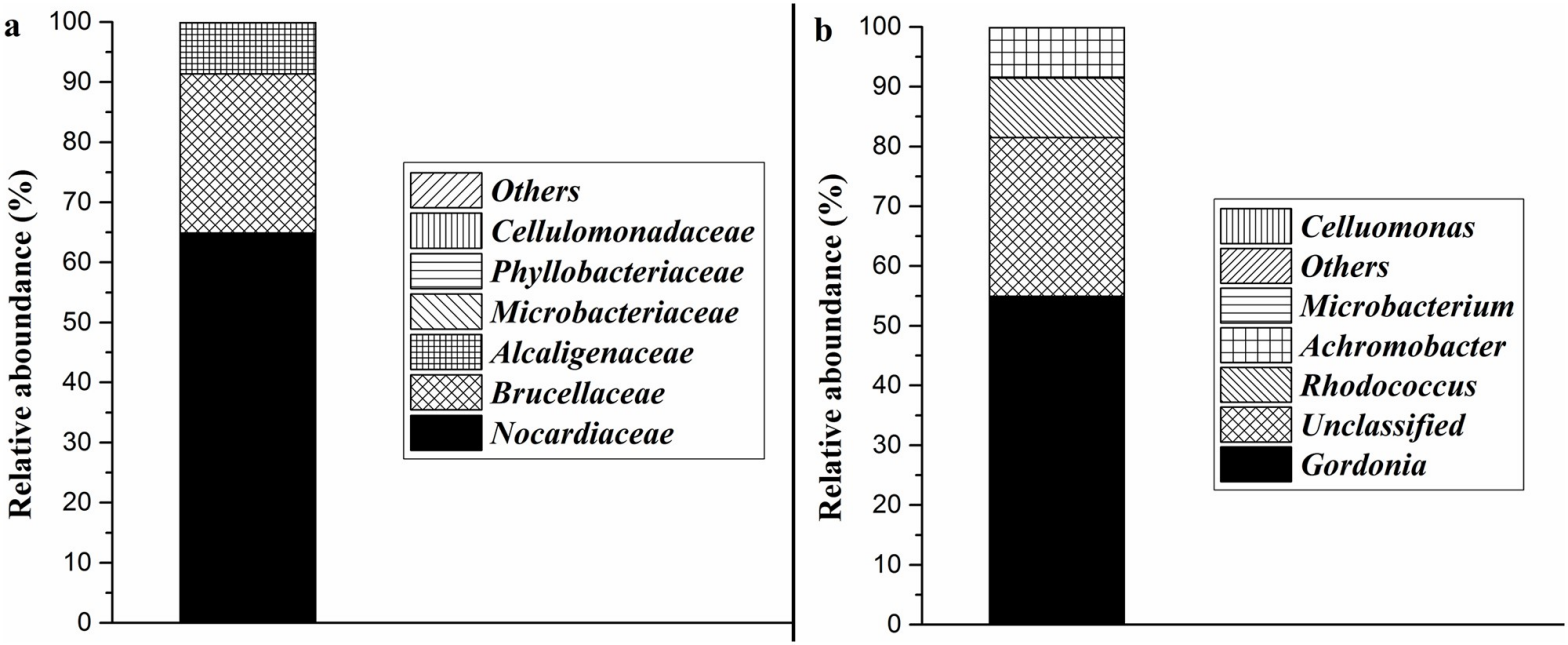
Bacterial community structure of LF. (a) at family level; (b) at genus level.

Community analysis revealed that LF was composed by about 7 families, and the mainly family were *Nocardiaceae* (64.86%), *Brucellaceae* (26.51%) and *Alcaligenaceae* (8.47%), the sum of the three family accounted for 99.84% of all reads (Fig 1a). Besides, the relative abundance of *Microbacteriaceae*, *Phyllobacteriaceae* and *Cellulomonadaceae*(0.01%) were 0.08%, 0.06% and 0.01%, respectively. At genus level, LF was composed by 7 genues and the main genus were *Gordonia* sp. (54.93%), *Rhodococcus* sp. (9.92%) and *Achromobacter* sp. (8.47%) (Fig 1b). The relative abundance of the genus of *Microbacterium* sp. and *Cellumonas* sp. were 0.08% and 0.01%, respectively.

Nowadays, there are many reports on the degradation of PAEs by *Gordonia* sp. and *Rhodococcus* sp. Jin et al. [37] studied the degradation effect of *Gordonia* sp. QH-11 on DBP and explored its degradation kinetics. Sarker et al. [38] studied the effect of *Gordonia* sp. Dop5 on the degradation of DOP. Wang et al. [39] investigated the effect of *Rhodococcus globerulus* WJ4 on the degradation of DEHP and Lu et al. [40] investigated the effect of *Rhodococcus* sp. L4 on the degradation of DMP, DEP and DBP. However, there are few studies on the degradation of PAEs by *Achromobacter* sp. Previous reports indicated that bacterial consortium is more suitable for bioremediation than pure bacteial strains, indicating LF is a more suitable bacterial consortium in bioremedaition of DEHP contamination under hypersaline environment [34, 35].

### Effects of initial pH value and temperature on DEHP degradation

**Fig 2:**
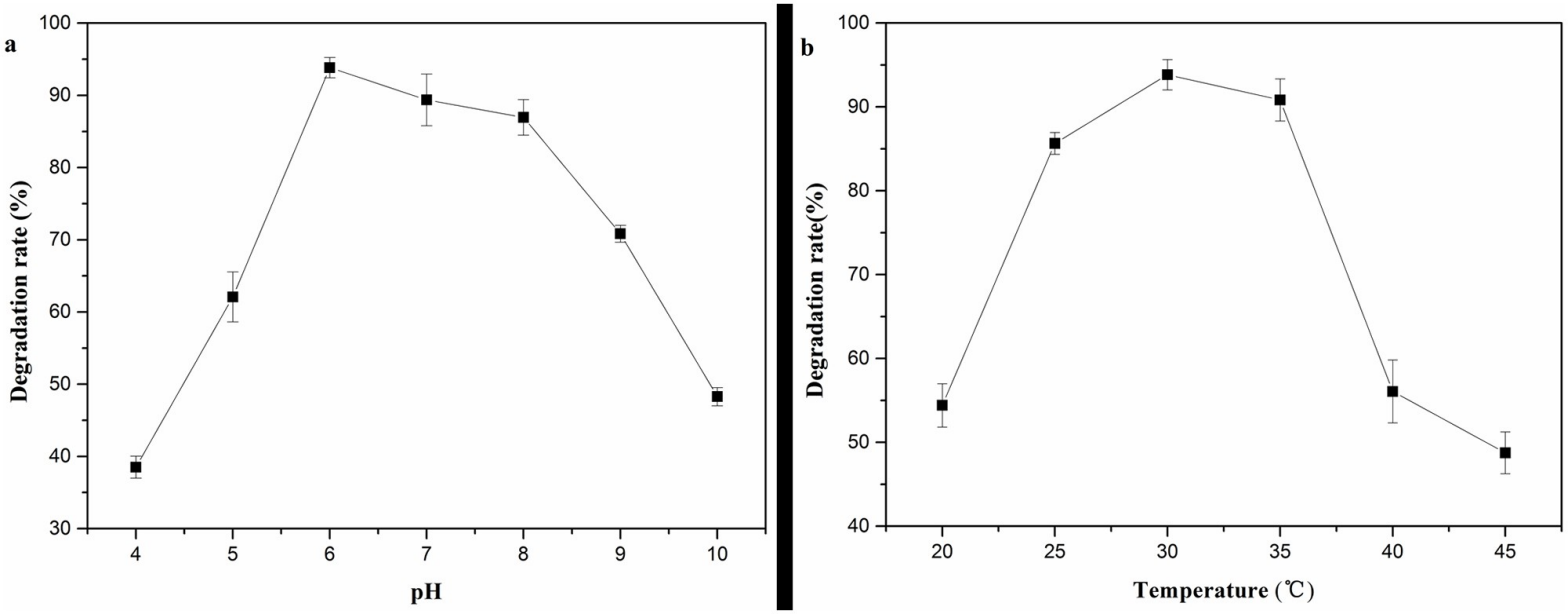
Effects of pH(a) and temperature(b) on DEHP (1000 mg/l) degradation by LF. Average values of three replicates are shown with the standard error of the mean as error bars.

Fig 2a illustrated the effects of initial pH on DEHP degradation by LF. The degradation ratio increased drastically (from 38.52% to 93.84%) when the initial pH of the medium increased from 4.0 to 6.0, and the highest DEHP degradation ratio (approximately 93.84%) was achieved at pH 6.0. In general, LF exhibited higher degradation ratio under neutral pH conditions (6.0–8.0), with all exceeding 87%. A higher initial pH (higher than 8.0) resulted in a decline of the degradation ratio, and the degradation ratio dropped to about 48% with the initial pH 10.0. All the DEHP degradation ratios exceeded 60% with the initial pH ranging from 5–9, indicating that LF had a wide pH value in degrading DEHP. This result was consistent with the previous reports. The optimal pH for DBP degradation by the consortium LV-1 was 6.0 and either lower or higher pH would inhibit the degradation of the consortium [34].

Temperature is a significant parameter affecting microbial degradability. As shown in Fig 2b, the DEHP degradation ratio increased rapidly as the temperature increased from 20°C to 25°C. DEHP degradation ratio was higher than 85% with the temperature ranging from 25°C to 35°C, and the maximum degradation ratio was 93.84% with the temperature at 30°C. DEHP degradation ratio declined sharply with the further increase of the temperature, which decreased to 46.68% as the temperature increased to 45°C. However, the degradation ratio was more than 55% with the temperature ranging from 20°C to 40°C, indicating LF had a broad temperature in the degradation of DEHP.

### Effects of NaCl concentration on DBP degradation

**Fig 3:**
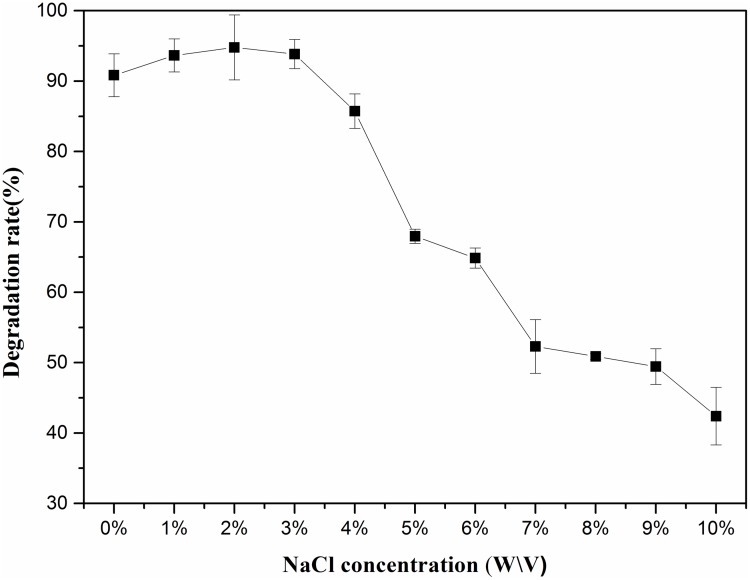
Effects of NaCl concentration on DEHP degradation by LF. Average values of three replicates are shown with the standard error of the mean as error bars.

The influence of NaCl concentration on DEHP degradation by LF was shown in Fig 3. The degradation ratio of DEHP was higher than 91% with the salinity concentration ranging from 0% to 3%, and the degradation ratio reached 86% with the salinity concentration at 4%. In general, the average salt content and temperature of sea water is about 3% and 25 °C [41], and the degradation ratio of DEHP by LF at 25 °C is 85.65%, indicating LF is well suitable for remediation the DEHP pollution in sea water. But a higher salinity concentration (5% to 10%) resulted in the decrease of the DEHP degradation ratio.

Salinity is an important factor affecting microbial growth and pollutant degradation, but there are just few halotolerant strains or consortium in degrading PAEs have been published in previous reports. *Burkholderiacepacia* DA2 was isolated by Wang et al. [31] from marine sediments for degradation of DMP, which could tolerate a salt concentration from 0% to 1%. The halotolerant bacterial strain *Sphingobium* sp. TJ was isolated from water of the Haihe Estuary by Jin et al. [32], which could degrade DBP with the salinity ranging from 0% to 4%. As far as we know, no study about biodegradation of DEHP by halotolerant bacterial consortium has been published yet. In the present research, DEHP degradation ratio by LF maintained higher than 50% within 48 hours with the salinity ranging from 0% to 9%. Such a superior halotolerant ability and high degradation efficiency of LF made it a potential application in bioremediation of DEHP in saline environment.

### Effect of inoculum size on DEHP degradation

**Fig 4:**
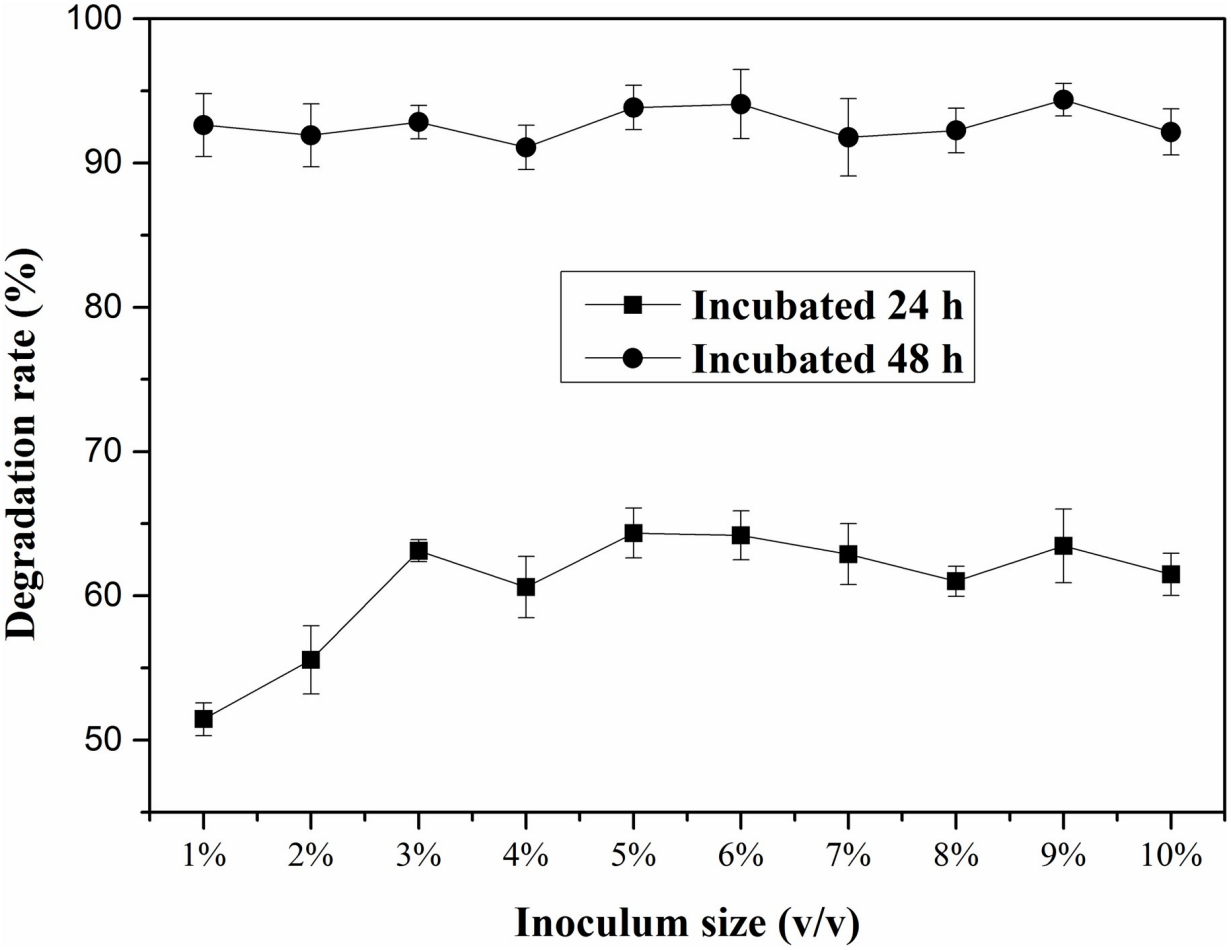
Effects of inoculum size on DEHP degradation by LF. Average values of three replicates are shown with the standard error of the mean as error bars.

The effects of inoculum size on DEHP degradation ratio are shown in Fig 4. Within 24 h of incubation, the degradation ratio of DEHP increased slowly with the increase of inoculum size and there was nearly no DEHP degraded in the control experiments (the inoculum size at 0%). The degradation ratio was about 51.45% at the inoculum size of 1%, and it gradually increased with the increase of the inoculum size. When the inoculum size was higher than 5%, the degradation ratio reached approximately 64% without apparent fluctuation. However, when the incubation time increased to 48 h, the influences of inoculum size on the DEHP degradation ratio were not significant, indicating that the inoculum size had little influence on DEHP degradation ratio with an incubation time ≥ 48 h. Based on the results of previous studies [42, 43], an inoculum size of 5% was used in the following experiments.

### Kinetics of DEHP degradation by LF

**Fig 5:**
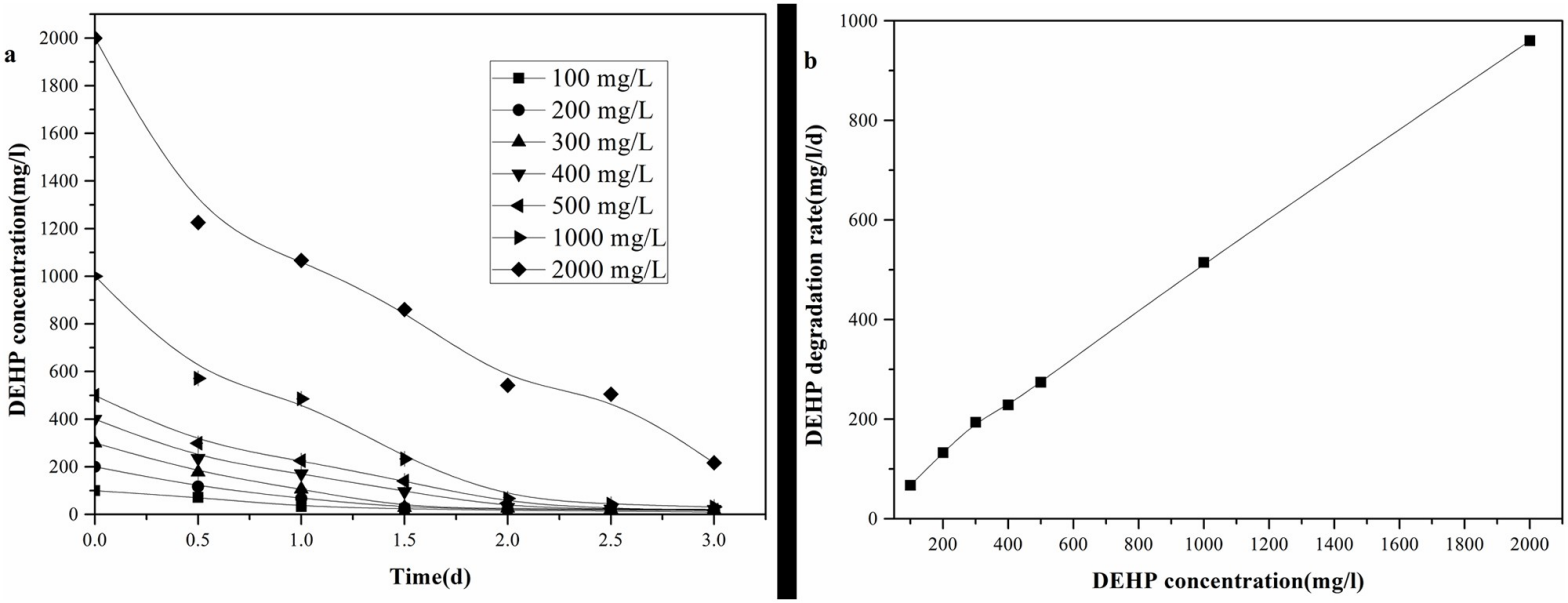
DEHP degradation kinetics (a) DEHP degradation; (b) DEHP degradation rate on the first day.

The degradation of DEHP by LF at different initial concentrations (from 100 to 2000 mg/l) was investigated, with the dynamic curves of DEHP concentration shown in Fig 5a. The degradation ratio of DEHP by LF was more than 93.84% within 2 days except at the initial concentration of 2000 mg/l. However, when the incubation time increased to 3 days, the degradation ratio reached 96.89% and 92.80% at the initial DEHP concentrations of 1000 and 2000 mg/l, respectively. The advantages of LF in degrading DEHP are obvious compared with previous reports. Bacterial strain MT-O has been isolated by Zhao et al. [44] for DEHP degradation. They found that MT-O could degrade DEHP with the initial concentration ranging from 50 to 200 mg/l, and a higher initial concentration (400–1000 mg/l) resulted in the decrease of the degradation rate. Xu et al. [19] studied the effects of *Acinetobacter* sp. SN13 on DEHP degradation. No lag phase was observed when DEHP concentration was lower than 400 mg/l, but the inhibitory effect appeared and the cell growth also started to decrease as DEHP concentration was higher than 400 mg/l. Therefore, LF is more suitable for bioremediation of DEHP contamination.

Fig 5b shows the degradation rate of DEHP by LF within the first day. The degradation rate of DEHP by LF increased from 67.25 to 960.10 mg/l/d with the initial concentration increasing from 100 to 2000 mg/l, indicating that higher initial concentration of DEHP did not exhibit inhibitory effects on the degradation ability of LF. Kinetics analysis revealed that biodegradation of DEHP by LF fitted well to the first-order reaction model (R^2^ ranging from 0.905 to 0.964), and the biodegradation half-life (t_1/2_) of DEHP varied from 3.61637 to 4.35519 days (Table 1). These results showed that LF could degrade DEHP efficiently with various initial concentrations.

**Table 1:** Degradation kinetics of DEHP by LF.

Initial concentration (mg/l)	Kinetic equations	t_1/2_(d)	R^2^
100	*Ln* C = - 0.04177t + 4.71629	3.86972	0.93516
200	*Ln* C = - 0.04691t + 5.31472	3.75267	0.96373
300	*Ln* C = -0.05376t + 5.83304	3.61637	0.95480
400	*Ln* C = -0.04225t + 6.01379	3.85730	0.95959
500	*Ln* C = -0.04042t + 6.24018	3.90158	0.94687
1000	*Ln* C = -0.04219t + 6.93578	3.85872	0.90490
2000	*Ln* C = -0.02568t + 7.49031	4.35519	0.91507

### Analysis of DEHP degradation intermediates

**Fig 6:**
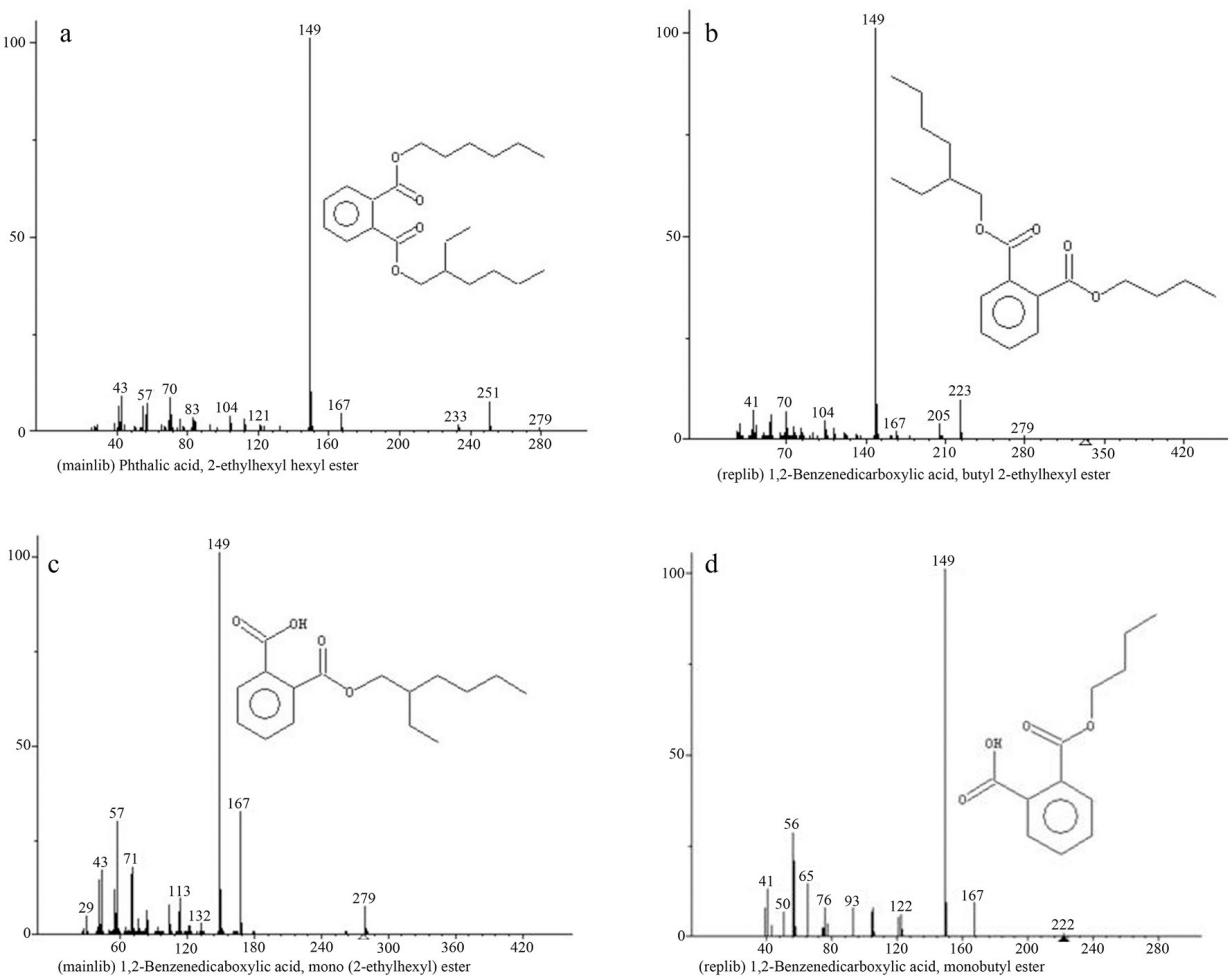
MS spectra of the intermediates of DEHP by LF. (a) 2-ethylhexyl pentyl phthalate (EHPP) (b) butyl (2-ethylhexyl) phthalate (BEHP) (c) mono-ethylhexyl phthalate (MEHP) (d) mono-butyl phthalate (MBP).

**Fig 7:**
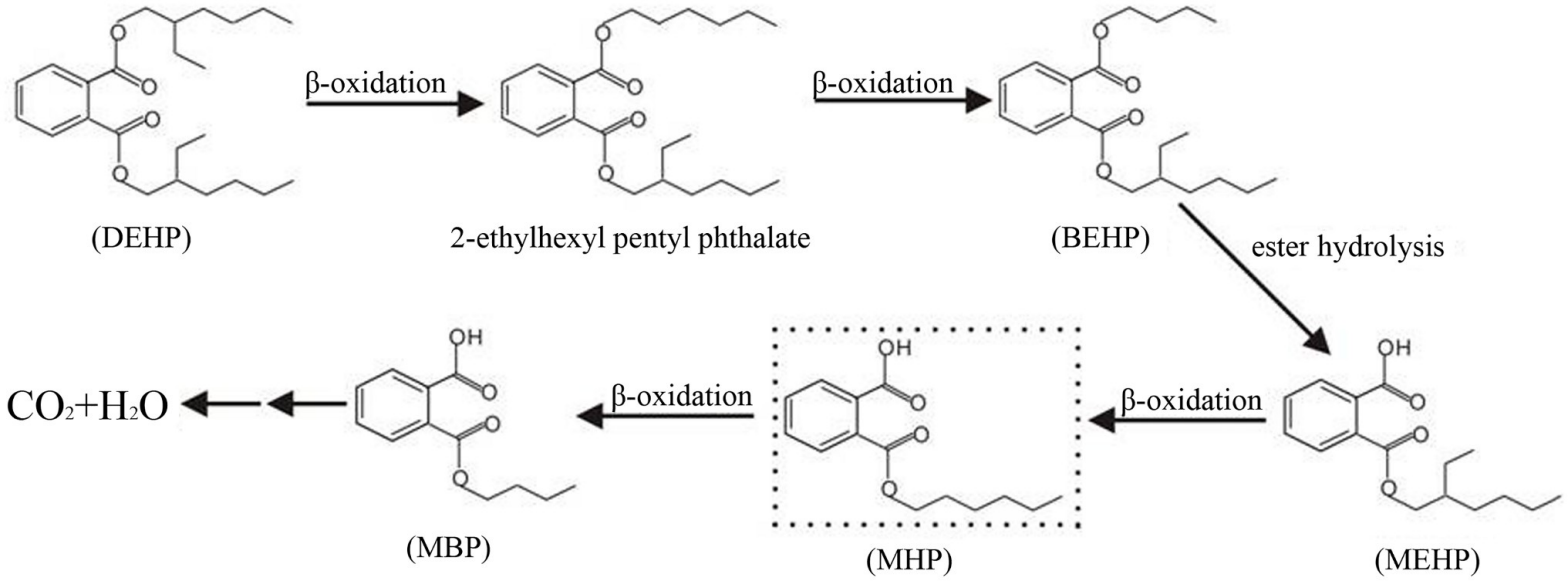
Proposed biochemical degradation pathway for DEHP by LF. The dashed box indicates the inferred intermediate not detected in this study.

The degradation intermediates of DEHP by LF were determined using GC-MS. A total of four major intermediates (2-ethylhexyl pentyl phthalate (EHPP), butyl (2-ethylhexyl) phthalate (BEHP), mono-ethylhexyl phthalate (MEHP), mono-butyl phthalate (MBP)) were discovered in the degradation process (Fig 6). The existence of EHPP and BEHP suggests the biodegradation of DEHP might be through β-oxidation. The ethyl group was dropped from the branch chain of DEHP formed the EHPP, and then another ethyl group was dropped and formed BEHP. After that, BEHP was transformed to MEHP through ester hydrolysis, and the ethyl group was sequentially dropped and formed mono-hexyl phthalate (MHP) and MBP. However, the metabolite of MHP was not detected in this study, possibly because the concentration of MHP was below the detection limits or only a small amount of MHP was accumulated. In previous studies, DEHP was commonly degraded by de-esterification in two steps, the hydrolysis of DEHP to MEHP and then to PA [18, 45, 46]. But it was not discovered in present research, this is mainly due to the efficient degradation ability of LF to PA, which resulted in the concentration of PA below the detection limits. Pradeep et al. [47] and Shailaja et al. [48] also found the similar results during DEHP degradation, no PA was detected. However, four new substances (EHPP, BEHP, MHP and MBP) were found in our study, thus the DEHP degradation mechanisms was been further explored. These results suggest that the consortium LF degrade DEHP via a complex biodegradation pathway (proposed pathway in Fig 7).

### Substrate utilization texts

To test the substrate utilization range of LF, MSMs were supplemented with different PAEs (400 mg/l). As shown in Table 2, LF could degrade various PAEs ranging from the shorter alkyl-chains such as DMP, DEP, and DBP to longer ester chains such as DOP. The results indicated that the consortium LF was able to utilize a series of PAEs with short or long alkyl-chains. In addition, the consortium LF was also able to utilize the common PAEs degradation intermediate PA, which further explained why PA was not detected in the degradation process. The broader degradation range of the consortium LF may be due to the high microbial diversity, which can expand microbial survival in the environment. Therefore, the consortium has great potential applications for degradation of PAEs contaminated sites.

**Table 2:** Growth profile of LF on various of aromatic compounds.

	Substrates						
	Control	PA	DMP	DEP	DBP	DOP	DEHP
OD_600_	0	0.433±0.008	0.586±0.005	0.578±0.006	0.541±0.003	1.239±0.009	1.172±0.002

## Conclusions

In this paper, a halotolerant consortium can utilize DEHP as the sole source of carbon and energy was enriched. The community composition was analyzed by Illumina sequence. The influences of pH, temperature, salt content, inoculum size and initial DEHP concentration on DEHP degradation by LF were determined. The intermediates of DEHP biodegradation were analyzed using GC-MS. This is the first report on DEHP degradation by halotolerant consortium, suggesting that LF poses great potential in remediating DEHP contamination.
